# Trends in coverage of hygiene and disease prevention topics across national curriculum frameworks for primary science, physical education, and health

**DOI:** 10.1007/s11125-020-09525-7

**Published:** 2021-01-20

**Authors:** Daniel Morrish, Marc Neesam

**Affiliations:** Cambridge Assessment International Education, The Triangle Building, Shaftesbury Road, Cambridge, CB2 8EA UK

**Keywords:** Covid-19, Science, Health, Hygiene, Primary, Curriculum, Physical education

## Abstract

The response to the Covid-19 pandemic raises a question about the role of national curriculum frameworks in acquiring and applying knowledge about hygiene and prevention of disease. For curriculum designers, this means determining what children of different ages should learn about these topics and how they should develop and apply this knowledge. Curriculum designers must do so amid trends towards reducing curriculum content and transitioning to competency-based curricula with transversal elements. Arguments can be made for placing health literacy competences, knowledge, and skills across the intended curriculum for science, physical education, and health. These are different disciplines with different models of knowledge, learning, and progression. This exploratory study looks at the placement of public health-related content in a selection of recently reformed, competency-based national curriculum frameworks from Europe, Africa, the Middle East, and Australasia. From these examples, it highlights risks and opportunities for incorporating public health messages into the intended curriculum.

National curriculum frameworks are policy documents that describe the relationship between national strategies for developing economic and human capital, education policy, and the aims and objectives of the curriculum. They normally include the structure of the curriculum (i.e., what should be learnt at various stages of education) and often feature statements of transversal skills and competences (UNESCO 2013).

A common response to societal challenges, such as those arising from the Covid-19 pandemic caused by the SARS-CoV-2 virus, is to propose amendments to these frameworks. For example, there have been calls for adding first aid (Cardiff Times 2019), road safety (Road Safety 2016), Wikipedia editing (Smulian 2019), and chess (Gurney Read 2014) to the frameworks used in the United Kingdom. The desire to place new content within these frameworks is intuitive; it will ensure that future generations are better prepared or more resilient with the knowledge, skills, attitudes, and values needed to thrive in a changing world (OECD 2018).

The challenge for curriculum designers and developers is responding to calls for new content and competences without increasing the curriculum load. Concerns about curriculum load have driven recent curriculum reforms across the world, summarised thus by the OECD: “Confronted with the needs and requests of parents, universities and employers, schools are dealing with curriculum overload. As a result, students often lack sufficient time to master key disciplinary concepts […]” (OECD 2018). To reduce curriculum overload, countries have used reform programmes to reorganise and reduce the content of their national curriculum frameworks, often embedding competencies and integrating subjects (NCCA 2010; Voogt and Roblin 2012; Nordin and Sundberg 2016).

## Curriculum models for science and health education

National curriculum frameworks for science education often seek a balance between educating the scientists of the future and developing a scientifically literate population (The Royal Society 2014). Education systems around the world already aim to produce scientists who can research and discover solutions to pandemics, technicians who can develop and deliver solutions, and members of the public who understand what is required of them and can distinguish between scientifically rigorous advice and speculative misinformation (Paakkari and Okan 2020).

There are intimate connections between the substantive knowledge of science and health curricula, which can make the relationship between them appear straightforward. For example, there is a scientific basis to the behaviours children develop in relation to hygiene and nutrition, applying microbiology to understand disease control. These apparent relationships are appealing to curriculum developers as opportunities to reduce load and increase coherence between disciplines (Zeyer 2012).

The placement of curriculum content needs to take account of discipline-specific learning progressions, the sequenced network of related learning needed to access and achieve competence (Heritage 2008). Progression is made evident in intended curricula by defining, in varying degrees of detail, increasingly sophisticated applications of knowledge and skills. In science, learners often progress from contextualised observations of the world to counterintuitive understandings of abstract concepts with increasing depth or breadth (Harlen 2015). In physical education, learners progress through increasing intensity, complexity, and strategy appropriate to their own physical development. In health education, learners progress by expanding their awareness of themselves, others, and the interrelationships they have with societal norms and practices (Nutbeam 2008). Therefore, cross-disciplinary curriculum content aimed at preparing learners for future pandemics needs to align with the progressions in understanding scientific concepts, awareness of healthy behaviours, and skills to organise oneself and others. This makes it important to understand the nature of the health messages being placed in different curriculum subjects and the impact of where they are placed.

## Aims and method

Changes made to curricula frameworks based on partial understanding of the issues may create new problems rather than remedying existing ones (Cambridge Assessment 2017). Our aim in this exploratory study is to identify current approaches to the placement of health messages in the competency-based curriculum frameworks used by the researchers in their professional practice. We hope that these descriptions can inform the response of curriculum developers to calls for review and modifications following the Covid-19 pandemic, by providing insights into the differences in placement between contexts and subjects/learning areas.

Our study is based on an analysis of several national curriculum framework documents. The document analysis approach enables the compilation of detailed descriptions of phenomena, in order to generate understanding and insight (Bowen 2009). From the text in these documents, we identified statements that could be associated either with public health and hygiene or with scientific understanding of disease transmission. However, content specific to language development was excluded, to avoid bias towards content aimed at learners whose first language is English. This minimised the risk that content was missed due to assumptions about the placement of health messages. In the context of the Covid-19 pandemic, statements were defined as “health” based on their relevance to communicable disease. They were also defined by the stage or grade at which they should be learnt. This enabled insights into the intended progression in applying knowledge and skills. Through comparison and contrast of salient observations, we were able to bring together reflections and develop a consensual evaluation of the main messages from the study.

## Selection of national curriculum frameworks for this study

The selection of documents for analysis is a potential limitation of the document analysis approach selected for this study. We selected five national curriculum frameworks that are publicly available in English, Estonia, Ghana, Kenya, Kuwait, and Australia. All five were part of our recent professional activities. Their common features include a competence-based approach, the definition of “general” or transversal competences, and the identification of cross-curriculum themes. These documents can therefore be said to be representative of current trends in national curriculum framework design (Voogt and Roblin 2012; Nordin and Sundberg 2016). Table 1 demonstrates that the five countries have different levels of health and educational investment and outcomes, along with spanning a broad geographical range.

**Table 1:** Education and health system indicators from the frameworks’ countries of origin

	Estonia	Ghana	Kenya	Kuwait	Australia
Primary education expenditure per capita	$6,327 ^1^	$ 142 ^2^	$ 140 ^2^	$6,335 ^2^	$9,546 ^1^
Health care expenditure per capita ^3^	$1,185	$68	$66	$1,068	$5,002
Life expectancy at birth ^3^	77.8	63.4	66.7	74.8	82.9
Mortality rate due to exposure to unsafe water, sanitation, and hygiene per 100,000^3^	< 0.1	18.8	51.2	< 0.1	0.1

^1^OECD 2020

^2^Calculated from proportion of GDP per capita spent on primary education (UIS 2014)

^3^World Health Organization 2019

The selected frameworks have different structures, so the following specific documents were included in the study.

- Estonia: General provisions of national curriculum for basic schools, Appendices 3–13 (Basic schools)
- Ghana: National Pre-tertiary Education Curriculum Framework, curriculum documents for lower primary and upper primary (excluding English, French, and Ghanaian language)
- Kenya: Basic Education Curriculum Framework, curriculum designs for Grades 1–4 (Volumes 2–4)
- Kuwait: Kuwait National Curriculum Framework, Kuwait National Curriculum Primary Education Curriculum and Standards (limited to physical education, science, and social studies)
- Australia: Content from the F-10 curriculum website (excluding English and language learning areas).

**Case 1: Ghana**

The National Council for Curriculum and Assessment (NaCCA) in Ghana recently revised its National Pre-Tertiary Education Curriculum Framework and the subject curricula to reflect a “standards-based” approach, which includes the incorporation of six cross-curricular, core competences and four cross-cutting and topical issues (NaCCA 2018, 2019). One of the cross-cutting issues is explicitly focused on health and hygiene, *Reproductive health and sanitation*.

The substantive and disciplinary knowledge and skills relevant to hygiene and disease transmission can be found in the Science Curriculum for Primary Schools (Basic 1–6). *Personal hygiene and sanitation* and *Diseases* run as sub-strands throughout the curriculum as part of a *Humans and the environment* strand. The content standards are repeated across all of B1–6: “recognise the importance of personal hygiene” and “know common diseases of humans, causes, symptoms, effects and prevention”. New curriculum standards are added at Basic 5, when learners are expect to “identify, discuss and appreciate the natural and human features of the environment and the need to keep the environment clean”. The progression in learning is defined by learning indicators associated with each of the content standards at each grade. This progression is illustrated in Table 2.

**Table 2:** Progression in indicators associated with content standards for the sub-strands *personal hygiene and sanitation* and *diseases* in the Science Curriculum for Primary Schools in Ghana

Grade	Personal hygiene and sanitation	Diseases
Basic 1	Explain the need for bathing and know how it is done Know the need for and how to clean the teeth Demonstrate understanding of the need for and how to wash the hands Know that clean air and water are essential to human health	Identify some common diseases that affect the skin and their causes
Basic 2	Explain how to keep the body clean and describe why it is important Know the need for keeping classrooms and school compound clean	Identify causes and prevention of ringworm Name some common water-borne diseases and their prevention
Basic 3	Describe ways of keeping the environment clean	Know how common skin diseases can be prevented Explain the term air-borne diseases and give examples
Basic 4	Know how to care for one’s self and the environment Describe ways of sustaining the environment through waste management	Identify causes, symptoms and prevention of measles Demonstrate understanding of the causes, symptoms and prevention of food-borne diseases
Basic 5	Know why it is important to wash clothes regularly Know how to keep washrooms clean Demonstrate how to clean the environment regularly	Explain the causes, symptoms and control of chicken pox Identify causes, symptoms and prevention of cholera
Basic 6	Identify the causes and effects of foul body odour on humans and how it can be prevented Describe ways of minimising waste	Explain the causes, symptoms and prevention of Eczema Know how to prevent meningitis

The progression model is similar for the two sub-strands. In both, the basic principles are established early on in Basic 1. From there, the progression expands contexts rather than concepts. Students learn the importance of cleaning in increasingly broad contexts (before becoming more specific again in Basic 6) and they learn about the prevention and treatment of an increasing range of diseases. There is no evident progression of scientific understanding in either sub-strand. The significance of this design decision is highlighted by the more conventional progression in scientific understanding in other sub-strands of the *Science* curriculum such as *Living and non-living things*, which progresses from observation to description to grouping to classification to life processes. The decision to have a different progression model for health-related content appears deliberate and justified by the need to repeatedly return to the same basic messages, with a broader scope as learners age. In the context of the Covid-19 pandemic, it is notable that the curriculum includes air-borne diseases, specifically at Basic 3.

Other subject curricula have references to health education. The curriculum for *Our world, our people* deals with health in the context of rights and responsibilities. In the latter grades it has a specific focus on reproductive health and its social aspects. There are also references to learning relevant to hygiene and disease prevention more generally. In Basic 3, this includes explaining “ways of promoting personal hygiene and safety as a responsible citizen” as part of the *All about us* strand. Environmental safety, including cleanliness and sanitation, is included in Basic 4. Basic 6 includes a more specific content standard: “Demonstrate understanding of personal hygiene during adolescence”. The religious education curriculum also has a content standard in Basic 2 that intends for learners to identify ways to care for their body, including personal hygiene.

Content relating to nutrition and diet is also spread across the curriculum. The curriculum for *Physical Education* focuses on healthy diets, which avoids duplication in the *Science* curriculum. The *Science and industry* sub-strand (part of the *Humans and the environment* strand in the *Science* curriculum) includes food processing and preservation, with a focus on technology rather than hygiene. It is specified that micro-organisms should not be included. This is also referenced in Basic 6 of *Our world, our people*, which includes food safety with no specific reference to infection and disease.

The Kindergarten curriculum also includes references to health and hygiene. The strand *All about me* includes themes of *caring for the parts of my body* and *keeping my body healthy by eating good food and taking my vaccination* in Kindergarten 1, while *personal hygiene and caring for parts of the body* and *eating good food and taking my vaccinations to keep my body healthy* appear in Kindergarten 2. This content focuses on behaviours associated with hygiene and diet, or on the acquisition of language related to the body, diseases, etc. There are also references to scientific understanding, such as the importance of different food groups and comparisons of weight and length.

**Case 2: Kenya**

The Kenya Institute of Curriculum Development (KICD) is currently redesigning its national curriculum based on the Basic Education Curriculum Framework (KICD 2017, 2019a, b). Within this framework, good health is referenced as one of the eight national goals of education in Kenya: “Education should inculcate in the learner the value of physical and psychological well-being for self and others”. The KICD has developed specific “curriculum designs” for environmental, hygiene, and nutrition education in Grades 1–3, reflecting the importance of these subjects to the curriculum.

In the *Environmental* curriculum, the *Social Environment* strand starts in Grade 1 with a focus on cleanliness of the home and learners’ role in keeping the home clean (2.1.2b *participate actively in making the home environment clean*, and 2.1.2c *demonstrate willingness to keep the home environment clean*). This continues into Grade 2 with identical learning outcomes. In Grade 2, the *Environment and its Resources* strand covers the potential health risks of contaminated water (1.2.1d and 3.5.1c), whereas Grade 1 focused on preserving the water supply. This is continued in Grade 3, where the focus is on practical steps to make water clean and safe (1.2.1a-d). Grade 2 also introduces the importance of keeping animal housing clean. Disease is not explicitly mentioned, although “risk to the animals” implies this (3.2.1d). As with the Ghanaian example, in Kenya Grade 3 extends learning by expanding the scope. Cleanliness of the home in Grade 1 is expanded to cleanliness of markets in Grade 3 (2.1.1a-c). Clean water at home in Grade 1 is expanded to the protection of reservoirs in Grade 3. The importance of clean water is also emphasised in the *Hygiene and Nutrition* curriculum designs (for example, Grade 3, Strand 1, Sub-strand 1.6 is *Making water safe for drinking*).

The *Hygiene and Nutrition* curriculum design is separated into four strands of *Health Practices*, *Personal Hygiene*, *Food* (which includes healthy diet and food hygiene, as well as etiquette and shopping), and *Safety* (which focuses on prevention of accidents and injury). The two examples of progression in Table 3 demonstrate the design intentions behind health messages focused on increased autonomy, with personal application of repeated messages.

**Table 3:** Progression in learning statements from two strands of the Lower Primary Level Hygiene and Nutrition curriculum design (Kenya)

	Strand 1: Health practices	Strand 2: Personal hygiene
Grade 1	1.1 Healthy habits (a) identify health habits that prevent illnesses, (b) state the importance of practicing health habits to promote wellbeing of self and others, (c) practise health habits that promote wellbeing, (d) appreciate the importance of practising health habits to promote wellbeing of self and others.	2.1 Care of different parts of the body (a) name materials used to clean the different parts of the body, (b) mention the procedures used to clean different parts of the body, (c) use appropriate materials to clean different parts of the body, (d) clean body parts without wasting cleaning materials, (e) appreciate the importance of a clean body for personal hygiene.
Grade 2	1.3 Use of different rooms in a house e) mention the importance of keeping the various rooms in a house clean and tidy 1.4 Cleaning of utensils (a) mention reasons for cleaning utensils at home, (d) appreciate the importance of cleaning the utensils at home 1.6 Keeping water safe from contamination (d) state ways in which we can prevent water contamination.	2.1 Use and care of personal items (a) identify the items used for personal cleanliness, (b) give reasons why we should not share personal items, (c) state the procedure used when cleaning items for personal use, (d) clean personal items to promote cleanliness for self and others, (e) identify materials that can be improvised for personal use (f) appreciate the importance of caring for personal items to promote cleanliness for self and others
Grade 3	1.1 Healthy habits (a) mention healthy habits that promote our well-being, (b) state the importance of practising health habits for our well-being, (c) practice health habits that promote our wellbeing, (d) appreciate the importance of observing health habits for our well-being.	2.1 Prevention of parasites in and out of the body (a) name common external parasites found on the body, (b) identify common external parasite found on the body, (c) name common internal parasites found in the body, (d) identify common internal parasites found in the body, (e) mention the causes of external and internal parasites in the body, (f) mention the effects of parasite infestation to the body, (g) state the importance of personal cleanliness in preventing internal and external parasites, (h) practice personal cleanliness to prevent parasite infestation.

In Grade 4, the Kenyan curriculum designs switch from *Environmental* and *Health and Hygiene* education to *Science & Technology*, *Home Science*, and *Physical and Health Education*. In the *Science & Technology* curriculum design, the focus is on the selected science topics rather than health education. The digestive system is a focal point in the biology content and the importance of nutrition is covered in the *Physical and Health Education*.

Health education is primarily dealt with in the *Home Science* curriculum design, which includes a sub-strand about common illnesses with the following learning outcomes:

- identify common illnesses in the locality
- communicate when feeling unwell to others
- identify the causes of common illnesses in the locality
- identify healthy practices that prevent illnesses in the locality
- practice healthy measures that prevent illnesses in the locality
- appreciate the importance of healthy practices in promoting good health in the locality.

There is also a sub-strand about cleanliness in the home.

In Kenya there is an additional focus on maintaining and preserving resources alongside the health messages. This is reflected in references to practical applications of learning, through the inclusion of learning outcomes to reduce water pollution and create water filters (*Environment* 2.2, *Water pollution*). The *Physical and Health Education* curriculum design (following on from the *Movement* curriculum design in Grades 1–3) also picks up the theme of clean water (Grade 4, 5.9, *Food intake during games and sports*).

Cutting across all learning areas in the Kenya curriculum are several pertinent and contemporary issues (PCIs) which have been integrated with the intention of providing opportunities “for learners to develop and apply their skills and knowledge, or in other words, their competencies” (KICD 2019a, b). The PCIs for health-related issues cover HIV/AIDS, substance abuse, and lifestyle. Within the lifestyle issues section, there are expected outcomes related to healthy living and personal hygiene. The curriculum designs for each learning area provide links to PCIs for each sub-strand, such as links to time taken on tooth brushing in mathematics, items which should and should not be shared with “My family” in Christian Religious Education, safe treatment of wounds in movement activities (with a specific link to HIV/AIDS), and preventing disease transmission through cleaning musical instruments in creative activities. An example of how PCIs progress is evident in the “communicable diseases” strand, which has links to strands covering HIV/AIDS. Learners are expected to progress from food hygiene (washing hands, consuming clean food and water) in pre-primary 1 to personal welfare (washing, toileting, avoiding cuts, immunisation, and deworming) in pre-primary 2. This is revisited in Grade 1 (washing, consuming clean food and water, and avoiding cuts) and extended in Grade 2 (clean environment, immunisation, and deworming). In Grade 3, the focus moves to other content such as water treatment, eye protection, and health nutrition.

**Case 3: Kuwait**

The Ministry of Education in the State of Kuwait published its first National Curriculum Framework in 2016 (Ministry of Education of the State of Kuwait 2016). It is a competence-based curriculum that describes the intended educational achievements in terms of “a coherent system of key, general and specific competences that are measurable by means of the learning achievement standards and their detailed indicators/ descriptors” (p. 10). There is no specific mention of health as part of the vision. However, there are references to health in two of the key competences which the national curriculum framework aims to develop. The definition of the *Personal development and learning to learn* competence includes “make informed choices and decisions about health, diet and physical culture and exercise”. By the end of primary education, learners who demonstrate this competence are expected to understand “the benefits of good health activities and applying these activities in their daily lives”.

This is expressed predominately in the *Physical and Health Education Curriculum and Standards*. Table 4 indicates that there is no significant progression in this curriculum content, as the competences for Grade 2 and Grade 4 are identical. What progression there is focuses on the sophistication of messages and their transmission.

**Table 4:** Progression of specific competences related to health in the Kuwait Physical and Health Education Curriculum and Standards

	Physical and Health Education Specific competences 1.3
Grade 1	Appreciating the value of basic motor skills, good nutrition and proper rest for good health
Grade 2	Appreciating the basic benefits of good nutrition and care of the body at home and when travelling to local communities
Grade 3	Sharing with excitement learning a new movement task by performing it with others in their class (Note: 3.1 Making use of a variety of information for improved personal and community health or safety in Kuwait)
Grade 4	Appreciating the benefits of regular physical activities, good nutrition, and proper care of the body at school, at home and in the community
Grade 5	Sharing information on the benefits of participating in physical activities (Note: 3.1 Exploring information available in brochures, Internet websites and other resources to develop good personal hygiene and good health practices. And 3.2 Training others with ideas and solutions to respond to global health problems)

The *Science Curriculum and Standards* also include health-related content; by the end of primary education, learners who are competent in science are expected to “formulate basic values and concerns relevant for their age group, associated with health, safety, care of nature and the environment”. In science there are explicit references to hygiene and disease prevention in curriculum (performance) standards for the general (science-specific) competence of “explaining and analyzing features, behavior, phenomena and processes in the living and non-living world by observation and guided interpretation”. These include:

- Identify and explain important hygiene and health needs for ourselves related to checking eyesight, care of teeth and vaccinations against diseases
- Formulate values associated with concerns for health, dietary issues, safety, care of nature and the environment, by:
- Describe what it means to be healthy related to the sense organs and indicate socially acceptable ways of behaving, eating and drinking
- Describe possible actions on ways to take care of plants and animals and keeping the environment clean in the neighbourhood
- Indicate valued ways of taking care by recognizing the need for clean air
- Indicate valued ways of taking care by recognizing the need for preserving the environment (terrestrial and marine)
- Recognize and illustrate the value of health care, the role of the World Health Organization, and preserving eco-systems. (Ministry of Education of the State of Kuwait 2016)

As with the examples for Kenya and Ghana, there is an association here between personal health and environmental health. The association is presumably intended to encourage learners to appreciate the dependency of clean water, air, food, and surfaces for maintaining their own good health.

In the context of the Covid-19 pandemic, it is interesting to note the inclusion of explicit references to vaccination (at the time of writing, vaccines for Covid-19 are waiting for approval) and the World Health Organisation. These are both unique in the national curriculum frameworks studied. The achievement of performance standards related to vaccination is achieved through the study of health guidelines, reading personal vaccination booklets, and visits to vaccination departments to look at vaccinations required for international travel. In other subjects, “health” is included as one of the “Facilities and Services of My Country” in the content of the *Social Studies Curriculum and Standards*.

The Kuwaiti curriculum is also distinct from the examples of Kenya and Ghana in that it incorporates health content in a conceptual progression. Learning about health is concentrated in Grades 1 and 4. Table 5 illustrates the progression in the specific competences, within the range of realities and personal values associated with the general competence, “Explaining and analyzing, features, behavior, phenomena and processes in the living […] world”. The range of realities includes health and hygiene within a progression that starts with learners identifying functions of their own bodies, moves to distinguishing and grouping living/non-living things, and ends with application of this knowledge in requirements for space travel. The range of personal values establishes the behaviours associated with health before progressing to concepts of environmental health. Again, the progression ends with the application of knowledge about healing in the context of space travel.

**Table 5:** Specific competences relating to a range of realities and range of personal values in the Primary Education Science Curriculum and Standards for Kuwait

	Range of realities	Range of personal values
Grade 1	1.1 Recognizing and identifying features of ourselves and their function 1.2 Identify sources of sound, light and heat in the home/school and their usefulness	1.4 Indicating and demonstrating safe healthy and socially acceptable ways of behaving
Grade 2	1.1 Identifying and comparing living and non-living things	1.3 Demonstrating how to care for plants and animals and the environment around us
Grade 3	1.1 Identifying and describing characteristics of fish allowing them to function in a marine environment 1.2 Identifying and describing characteristics of birds allowing them to function in the Kuwaiti environment	1.4 Identifying and valuing healthy air 1.5 Identifying and valuing preserving the Kuwaiti environment
Grade 4	1.1 Identifying and explaining important features of hygiene and health care	1.4 Valuing the need for caring about our health and preserving eco-systems
Grade 5	1.1 Identifying and explaining features of ourselves and ecosystems important for being in space	1.3 Valuing the body’s healing abilities and appreciating creativity in the designing of ecosystems in space

Learners also learn to identify the needs of living things (Grade 1), classify animals by diet (Grade 2) and identify nutritional and unhealth foods (Grade 3).

**Case 4: Estonia**

The Estonia national curriculum for basic schools was published in 2014 (Government of the Republic of Estonia 2014). It is competence-based with general and subject-based competences. The eight general competences to be developed include: “self-determination competence – the ability to understand and evaluate oneself, one’s weaknesses and strengths; analyze your behavior in different situations; behave safely and follow a healthy lifestyle; solve communication problems”. “Competence in mathematics, science and technology” includes making evidence-based decisions and understanding the importance and limitations of science and technology. Basic school graduates who are competent in science are expected to “value the environment as a whole; undertake a responsible and sustainable lifestyle connected to the environment and follow a healthy way of life”.

In Estonia, the government regulation for the national curriculum for basic schools also describes eight cross-curricular topics which “span numerous subjects, and fields that are priorities for society”, including *health and safety*. It includes no explicit references to disease prevention at the level of definition provided in the regulation. However, it describes broader aims for the student to “develop into” a “healthy member of society who is capable of following healthful lifestyles”. As with the previous examples this is linked to concepts of a healthy environment, and the regulation mandates that basic schools will ensure “opportunities for promoting physical education and healthful lifestyles within and outside of lessons”.

Appendix 13 to the regulation provides a progression in the cross curricular topics. *Health and Safety* progresses from common healthy behaviours, across the first six grades of basic education, to values associated with these behaviours in the third stage of basic education. This limited progression can be contrasted with the progression in the cross-curricular topic of *Environmental and Sustainable Development*, which progresses with an expanding scope using a model like those used in previous cases. Table 6 demonstrates how, starting from direct personal experience, understanding progresses to home and national issues, before moving to global issues and reliance on natural resources in the third stage of basic education.

**Table 6:** Comparison of progression in competence in two cross-curricular topics from the Estonian national curriculum

	Health and safety Competence	Environmental and Sustainable development Competence
First stage	Maintains cleanliness and order, looks after his or her appearance and health and has a desire to be healthy.	Acts in a prudent manner with regard to nature.
Second stage	Maintains cleanliness and order, looks after his or her appearance and health and has a desire to be healthy.	Values a sustainable lifestyle, is capable of asking questions in the field of natural sciences and obtaining information on natural sciences, knows how to act in nature, takes an interest in nature and exploring nature.
Third stage	Values and follows a healthful lifestyle and is physically active.	Understands the interrelations between man and environment, takes a responsible attitude to the environment and lives and acts in an environmentally sustainable manner. Knows how to pose natural science questions, discuss them, present scientific positions and make conclusions on their basis.

The relationship between the cross-curricular topic *Health and Safety* and the science curriculum is set out in the Appendix to the regulation, which describes the expected learning in Natural Science (Appendix 4): “The theoretical basis for suitable health behaviours is primarily established in Biology and Chemistry lessons”. The theoretical basis is set out in a strand of learning specifically focused on *Human Beings* and in the third stage in *Structure and Living Processes of Microorganisms*. In this way, the health content of the Biology curriculum follows a conceptual progression model common in science curricula. Learners focus first on observable phenomena (external features and behaviour) before moving onto internal systems, and from there to microscopic organisms and interdependencies.

Hygiene is included in the content of the first stage of study, where is it combined with a basic understanding of external features of the human body and the relationship between people and their environment. Hygiene is not mentioned in the second stage, which focuses on the internal structures of the human body (foundational learning for deeper understanding of health) and classification of living things (including bacteria and their importance to humans, an implicit reference to disease prevention). Health is mentioned in the second stage of education in the context of lifestyle and diet. In the third stage of study, as part of the biology content, the strand on *Structure and Living Processes of Microorganisms* includes “know how to prevent the most common human bacterial and viral diseases and value healthy lifestyles”. The third stage also includes health in terms of various systems including skin, skeleton, circulation, digestion, breathing, sexual health, etc.

**Case 5: Australia**

The Australian Curriculum, Assessment and Reporting Authority (ACARA) introduced a new Australian Curriculum in 2014 (ACARA 2010). It is structured with learning areas (disciplinary knowledge, skills, and understanding), general capabilities (analogous to competences used in other curricula), and cross-curriculum priorities (the wider community priorities of Aboriginal and Torres Strait Islander Histories and Cultures, Australia’s relationship with Asia, and sustainability). One of the general capabilities is “Personal and Social capability”. The definition of this capability builds on the Melbourne Declaration on Educational Goals for Young Australians (MCEETYA 2008), which promotes the development of students who “have a sense of self-worth, self-awareness and personal identity that enables them to manage their emotional, mental, spiritual and physical wellbeing”. The learning continuum for this capability does not explicitly cite the knowledge, skills, and understanding required for health or hygiene; instead, it sets out skills and abilities that will enable learners to make good decisions. It is in the interaction with subjects such as Health and Physical Education and Science that students are encouraged to apply this capability to health and hygiene.

In the Australian F-10 curriculum there is no explicitly health- and hygiene-related content described in the Science learning area. It is all contained within the Health and Physical Education learning area. The elaboration statements in Table 7 show how practical disease prevention is established in early years, before the progression moves from personal hygiene and action to communication and increasing sophistication of the media used to share those messages.

**Table 7:** Elaborations in “Contributing to healthy and active communities” of the Health and Physical Education learning area of the Australian curriculum

Years	Elaboration
Foundation	Understanding the importance of personal hygiene practices, including hand washing, face washing, nose blowing and toilet routines
Years 1-2	Recognising how their actions help keep classmates safe, including identifying things not to be shared due to potential of contamination, infection and anaphylaxis
Years 3-4	Creating promotional posters to display around the school containing positive health and physical activity messages
Years 5-6	No explicit elaboration of disease (investigating practices that help promote and maintain health and wellbeing)
Years 7-8	No explicit elaboration of disease (investigating preventive health practices relevant to young people, and designing and implementing health promotion activities targeting these practices)
Years 9-10	No explicit elaboration of disease (creating and evaluating visual and multimodal health campaigns in print-based and digital environments to promote health and wellbeing in their community)

The progression in these elaborations can be contrasted with those in the *Science* learning area. In *Science*, the progression in the biological sciences follows the understanding of the requirements of life (Year 1), growth and reproduction (Year 2), life cycles (Year 4), adaptations (Year 5), impact of physical conditions on growth (Year 6), and cells (Year 8). The science progression here is conceptual and develops the scientific understanding and skills required to create increasingly sophisticated health campaigns.

## Discussion

Curriculum content relating to health and physical wellbeing is frequently placed in science education, physical education, or personal/social responsibility education, either as discipline-specific or cross-cutting content. Where there is a choice of subjects, learning areas, and transversal competences through which to address topics such as hygiene and disease prevention, curriculum developers need to decide how these topics should be included and what other content they should be associated with. This study of hygiene and disease prevention placement in five national curriculum frameworks with cross-cutting themes found five different ways for integrating health education content. This was an exploratory study to identify phenomena related to the positioning of health messages in different subjects/learning areas of recently published, competency-based national curriculum frameworks. Analysis of further national curriculum frameworks, including those not available in English, would help to triangulate these observations.

In the present study we offer a snapshot of current national curriculum frameworks. These documents will benefit from being stable over a long period of time to promote the development of high-quality teaching and learning programmes (Cambridge Assessment 2017). Analysis of less accessible obsolete frameworks is necessary to identify how the trend of competency-based frameworks with cross-cutting themes has influenced the placement of health messages. Paired with data from reliable assessments of learning, it would be possible to establish the relative impact of these different approaches. As four of the five frameworks reviewed here are relatively new, it may be some time before such analysis is possible, particularly since Australia is already commencing another curriculum review.

*Science education is universal; health education is context-specific*

Science and health education have a different balance of substantive and disciplinary knowledge. The examples of science curricula reviewed have common substantive knowledge (for example, the requirements of living things). The disciplinary knowledge is also common across all examples (for example, learners are taught to challenge, predict, and test theories) and is prominent in the curriculum, often providing an overarching framework for the substantive knowledge. These frameworks therefore reflect consensus on the priorities for the substantive and disciplinary knowledge of science (e.g., Harlen 2010; Mullis and Martin 2017).

There is more variation in the selection and integration of content related to public health and personal hygiene. The common conclusions, caveats, and confidence intervals of science are replaced with the contextual clarity, conformity, and certainty of health messaging. Ghana incorporates all health messages except reproductive health (cross-cutting theme) and diet (physical education) in its science curriculum. Kenya, Kuwait, and Australia all have dedicated learning areas focused on health. In Kenya and Australia, this is reinforced by cross-cutting themes related to health. Australia’s curriculum differs from Kenya’s and Kuwait’s in not having explicitly health-related content in the science curriculum. Estonia focuses health-related content in the interaction between science and cross-cutting themes. The nature and purpose of health content in these national curriculum frameworks is primarily concerned with substantive knowledge, which is often specific to the national context. The focus on clean water in Kenya, communication in Australia, and vaccination in Kuwait indicates very specific health messages that were not replicated in the curriculum frameworks of other countries. As this is a document review, we have not investigated the decisions behind these frameworks and are not aware of the extent to which the MERS outbreak in 2012 influenced this latter design decision.

In contrast to science education, in health literacy there is a recognized need for adaptation to local contexts (e.g., Rimal and Lapinski 2009; Zanchetta et al. 2012; Escoffery et al. 2018) and international consensus in the definition of health literacy is still developing (Lynch and Soukup 2016; Vamos et al. 2020). This emphasises the specific risk of “policy borrowing” in the context of health education. Risk levels may be different in countries such as Estonia, Australia, and others where levels of communicable disease are relatively low, and where the health messages related to lifestyle, nutrition and exercise are more consistent. The question of what health content should be included also highlights some interesting examples of curriculum content in the context of the Covid-19 pandemic. The *Air Pollution* sub-strand of the Kenyan curriculum design for Grade 4 Science & Technology includes a learning outcome to “make a functional dust mask using locally available materials”. Given the shortages of personal protective equipment during the Covid-19 pandemic, that may turn out to be a beneficial inclusion.

*Differences of progression in science and health education*

Progression in a curriculum is about the necessary (not always linear) steps towards deeper, broader, and more sophisticated understanding. The substantive knowledge that explains why behaviours are advantageous are usually introduced at later stages of education rather than when the behavioural expectation is developed. This is significant for the inclusion of public health messages in the curriculum, as the behavioural learning often develops in advance of understanding of the scientific basis for action. During the recent Covid-19 pandemic, the requirement to wash hands and socially distance needed to be understood and applied instantly by all age groups. All the curriculum frameworks reviewed demonstrated a focus on behaviour in early grades, while the science curriculum progressed learners’ understanding of living things to the point that bacteria and viruses could be introduced later.

*Beyond the curriculum*

A national curriculum framework is not the sole instrument for ensuring educational success and cannot ensure coherence on its own (Oates 2010). The national curriculum frameworks in this study have all been recently reformed and all contain much of the learning that would be considered relevant to primary school learners facing a future pandemic response (hygiene, etc). The specific role of vaccination is only covered in the Kuwaiti national curriculum framework. However, the national curriculum frameworks of Estonia and Australia offer enough flexibility for similar content to be included through implementation. In general, learning about hygiene is focused in the very earliest grades on healthy behaviours and habits. These countries are unlikely to require reform of the intended curriculum to ensure their learners have access to the knowledge and skills required to respond to future pandemics. Investment in teacher professional development has been identified as necessary to the success of teaching health literacy (Birch et al. 2019), and implementation of the curriculum may need to be adjusted to take account of specific responses to new diseases. For example, during the Covid-19 pandemic the Kenya Institute of Curriculum Development has worked with UNESCO to implement a digital health literacy programme on the Kenya Education Cloud, as part of the Education Sector Covid-19 Emergency Plan.

## Conclusion

The space in a national curriculum framework is finite and adding new content implies condensing other content to make space for it. While there is a common approach to the disciplinary knowledge of science, the selection and organisation of the substantive knowledge of science and health will often require local contextualisation to ensure that context relevant knowledge, skills, and competences are made available to all learners. This study describes how five different curriculum authorities who wished to include health messages in their curriculum have done so, showing that in primary education they are supportive of the learning progressions in both subjects rather than producing curriculum cul-de-sacs or add-ons.
